# A Semi-Quantitative Approach to Nontarget Compositional Analysis of Complex Samples

**DOI:** 10.1021/acs.analchem.4c00819

**Published:** 2024-11-07

**Authors:** Rhianna L. Evans, Daniel J. Bryant, Aristeidis Voliotis, Dawei Hu, HuiHui Wu, Sara Aisyah Syafira, Osayomwanbor E. Oghama, Gordon McFiggans, Jacqueline F. Hamilton, Andrew R. Rickard

**Affiliations:** † Wolfson Atmospheric Chemistry Laboratories, Department of Chemistry, University of York, York YO10 5DD, United Kingdom; ‡ Centre for Atmospheric Science, Department of Earth and Environmental Sciences, School of Natural Sciences, University of Manchester, Manchester M13 9PL, United Kingdom; ¶ National Centre for Atmospheric Science, University of Manchester, Manchester M13 9PL, United Kingdom; § National Centre for Atmospheric Science, University of York, York YO10 5DD, United Kingdom

## Abstract

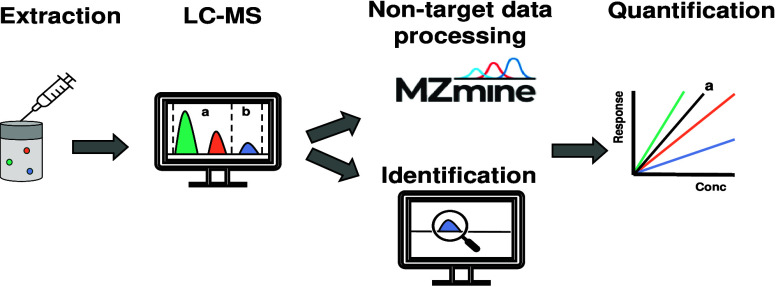

Nontarget analysis (NTA) by liquid chromatography coupled to high-resolution mass spectrometry improves the capacity to comprehend the molecular composition of complex mixtures compared to targeted analysis techniques. However, the detection of unknown compounds means that quantification in NTA is challenging. This study proposes a new semi-quantitative methodology for use in the NTA of organic aerosol. Quantification of unknowns is achieved using the average ionization efficiency of multiple quantification standards which elute within the same retention time window as the unknown analytes. In total, 110 authentic standards constructed 25 retention time windows for the quantification of oxygenated (CHO) and organonitrogen (CHON) species. The method was validated on extracts of biomass burning organic aerosol (BBOA) and compared to quantification with authentic standards and had an average prediction error of 1.52 times. Furthermore, 70% of concentrations were estimated within a factor of 2 (prediction errors between 0.5 and 2 times) from the authentic standard quantification. The semi-quantification method also showed good agreement for the quantification of CHO compounds compared to predictive ionization efficiency-based methods, whereas for CHON species, the prediction error of the semi-quantification method (1.63) was significantly lower than the predictive ionization efficiency approach (14.94). Application to BBOA for the derivation of relative abundances of CHO and CHON species showed that using peak area underestimated the relative abundance of CHO by 19% and overestimated that of CHON by 11% compared to the semi-quantification method. These differences could lead to significant misinterpretations of source apportionment in complex samples, highlighting the need to account for ionization differences in NTA approaches.

## Introduction

The ability to probe molecular composition has been revolutionized by liquid chromatography coupled to electrospray ionization (ESI) high-resolution mass spectrometry (LC-HRMS). LC-HRMS coupled with nontarget analysis (NTA) allows the detection of thousands of compounds present within complex sample matrices compared to a relatively small number of compounds (<100) in targeted analyses. For instance, in a targeted analysis of ambient particulate matter, Pereira et al.^1^ identified only 20 compounds which equated to less than 1.1% of the total mass, highlighting the significant advantages of using NTA. NTA approaches using LC-HRMS have previously been applied to detect emerging contaminants and hazardous substances in a range of complex samples such as environmental matrices and the food and drink industry.^1−4^ However, the quantification of unknown compounds remains challenging as traditional methods of calibration with authentic standards are not possible due to the lack of commercial availability and the sheer numbers of detected compounds.

For this reason, many prior NTA studies of complex samples use metrics such as peak area and the number of molecular formulas to convey the relative abundance of different compounds.^1,2,5−9^ However, the variability in the relationship between instrument signal and compound concentration means that this approach does not lead to accurate quantification.^10^ This phenomenon is a result of ionization efficiency, which is a measure of the ability of a species to ionize within the ESI source. Ionization efficiency is highly structurally specific and can vary by multiple orders of magnitude between different compounds including structural isomers.^10,11^ Additionally, the choice of ESI source, mobile phase, pH, and the percentage of organic modifier content across a gradient elution program could further affect the ionization efficiency.^12−15^ However, Kruve^12^ observed that generally ionization efficiency values were well correlated between methanol and acetonitrile mobile phases.

Recent efforts to quantify unidentified compounds have utilized machine learning to build predictive models of ionization efficiencies using physicochemical properties of analytes such as p*K*_a_, polarity, and the mobile phase composition.^11,12,16,17^ Alternatively, a second class of models predict relative ionization efficiencies (RIE), i.e., how well a species ionizes relative to a reference compound.^18−21^ Despite differences in the reference compound, the predictive RIE models developed by Bryant et al.,^18^ Mayhew et al.,^19^ and Liigand et al.^20^ perform similarly with R^2^ and the root-mean-square error (RMSE) ranging from 0.62 to 0.66 and 0.35 to 0.59, respectively. Furthermore, the model developed by Liigand et al.^20^ was constructed using data from a range of chromatographic conditions; however, little effect was observed on the model prediction accuracy. Application of the Liigand et al.^20^ model to quantify myotoxins and pesticides in cereals yielded a quantification error of 5.4, which is defined as the ratio between predicted concentration and the true concentration certified via an authentic standard. However, the main drawback of these models is the need to know the structure for quantification. In an NTA workflow, the number of structurally assigned compounds is usually low compared to the total number of detected compounds,^1^ which can be further impacted by the instrumental workflow and data quality across multiple samples. For instance, the use of data-dependent fragmentation mass spectrometry (ddMS^2^) will fragment the topmost abundant ions per scan. A more recent approach used fragmentation mass spectra (MS^2^) to obtain molecular descriptors for the prediction of ionization efficiency, allowing nonstructurally identified compounds to be quantified with an average prediction error, the ratio of predicted:true concentration, of 4.^22^ However, in data-dependent analysis used in 60% of NTA studies for environmental matrices,^23^ not all compounds will reach the threshold for subsequent fragmentation. Therefore, if relying on MS^2^ spectra for quantification, there can be a loss of compositional information. For example, Wang et al.^24^ observed in a typical nontarget workflow using data-dependent acquisition that only 39% of detected compounds have MS^2^ spectra, meaning the majority of data was discarded from compositional analysis. Using data-independent acquisition (DIA) can provide improved MS^2^ spectral coverage,^25^ and recent advances in DIA strategies such as SWATH-MS provide high quality, quantitation accuracy, and reproducibility.^26^ However, the data processing to deconvolute the DIA spectral output can be more challenging and time-consuming.^25^

Complete characterization of the molecular composition requires all compounds with and without MS^2^ spectra to be quantified. The analysis presented here uses a quantification methodology known as semi-quantification, where multiple proxy standards are used for quantification via surrogate calibration curves. In many semi-quantification studies to date, typically a singular structurally similar proxy standard is used.^27−36^ However, the selection of an appropriate surrogate is essential to reduce quantification errors.^37^ Reported semi-quantification errors, defined as the ratio of predicted:true concentration, can be as high as 10.^27,36,38,39^ The study of organosulfates in organic aerosol which are commonly used as tracers for secondary organic aerosol (SOA) has widely applied semi-quantification methods.^28−31,33−35,40^ For example, Li et al.^28^ suggested using camphorsulfonic acid as a surrogate standard for nitroxy organosulfates due to its similar structure. For C_2_–C_3_ organosulfates, multiple studies use glycolic acid sulfate as a proxy.^29−31^ However, this incorrectly assumes that all compounds of the same chemical class, in this case, organosulfates, ionize equally to that of a singular quantification marker. In reality, ionization in an ESI source is structurally specific, can increase with retention time,^41^ and can be affected by gradient elution due to changes in the mobile phase.^40^ Therefore, improved semi-quantification methods adopt closely eluting surrogate standards to the target compound,^32−34,38,42,43^ with reported prediction errors of 1.74–3.20 compared to quantification with authentic standards. However, the majority of semi-quantification studies using structurally similar surrogate standards were applied only to quantify a small subset of compounds (<10) and were quantified using a singular marker.^27−29,31,42^ In this study, we present a new semi-quantification method using 110 authentic standards and a series of retention time windows to derive scaling factors and uncertainty estimates from multiple proxy standards with a range of ionization efficiencies in each window. We then apply the method within an NTA of laboratory-generated biomass burning organic aerosol (BBOA) containing up to 2357 detected organic compounds in a single extract.

## Experimental Section

### Sample Collection

The newly developed semi-quantitative NTA methodology was used for detailed compositional analysis of BBOA from wood burning . The samples were taken from a series of wood burning experiments conducted at the Manchester Aerosol Chamber (MAC). The design and characterization of the MAC has previously been described in detail in Shao et al.^44^ In brief, the wood burning experiments aimed to investigate the impact of the burn phase, i.e., flaming and smoldering conditions, on the physical and chemical characteristics of the emitted aerosol. Particulate matter was sampled onto filters either at the flue of the wood burner for 5 min at 2 L/min or after an aging period under dark or light conditions inside the MAC at a flow rate of 3 m^3^/min for 4 min. Under dark conditions, no further oxidants were added to the chamber; therefore, it does not reproduce the chemistry of a nitrate radical (NO_3_) or ozone (O_3_) oxidation observed in the atmosphere at night. Instead, changes in the aerosol composition are likely driven by evaporation and in-particle chemistry. We define these 3 sample types per burn phase as fresh flue, dark aged, and light aged.

Quartz filters (Whatman QMA, 47 mm) were individually wrapped in foil and prebaked at 500 °C for 5 h prior to use. After collection, the filters were wrapped in the prebaked foil and then stored and transported at −20 °C for offline ultrahigh-performance liquid chromatography coupled to high-resolution mass spectrometry (UHPLC-HRMS) analysis at the University of York. Filters were extracted using methanol, and the full methodology for the filter extraction is provided in the Supporting Information.

### Instrument and Data Analysis

Filter sample extracts and authentic standard solutions were analyzed using an Ultimate 3000 UHPLC (Thermo Scientific, USA) coupled to a Q Exactive Orbitrap MS (Thermo Fisher Scientific, USA) with heated electrospray ionization (HESI) in negative mode. Authentic standard solutions, using compounds in Table S1, were prepared in mixtures of 50:50 MeOH:H_2_O with no overlapping of retention time between standards across the concentration range: 5, 2.5, 1, 0.5, 0.25, 0.125, and 0.0625 ppm. The wood burning samples were analyzed once by UHPLC-HRMS alongside solvent blanks and chamber blanks, taken from a clean chamber. The UHPLC-HRMS methodology is based on a well-characterized method developed by Bryant et al.^45^ and Pereira et al.^1^ for the exploratory compositional analysis of organic aerosol. Compound separation was achieved using a reversed-phase C_18_ 2.6 μm × 2.1 mm × 10 mm Accucore column and a mobile phase consisting of 0.1% (v/v %) formic acid (Acros Organics) in water (A, LC-MS Optima grade) and methanol (B, LC-MS Optima grade). These conditions enable the separation of a wide range of polar and nonpolar compounds,^46^ and the more acidic mobile phase can improve chromatographic retention and resolution as well as increase sensitivity.^47−49^ Full details of the UHPLC-HRMS method can be found in the Supporting Information. Spectra were acquired using XCalibur 4.3 (Thermo Scientific, USA) and analyzed using a nontargeted workflow developed in MZmine 2.53 and MZmine 3.9.0 software. Detailed NTA workflows are given in Tables S2 and S3. MZmine 2.53 software assigned molecular formulas to detected features, and MZmine 3.9.0 software enabled the identification of species via an in-house-built spectral library of authentic standards. The workflows were then merged for the remainder of the analysis. Post-processing of the MZmine output was achieved by (i) choosing the best formula predicted by MZmine 2.53, (ii) performing a blank subtraction, and (iii) removing duplicated data. Formula predictions were allocated providing the following criteria were met: 0.5 < H/C < 3.0, 0.05 < O/C < 2.0, N/C < 1.0, S/C < 0.5, and Cl/C < 0.2. The formula with the lowest mass tolerance in ppm was then selected as the “best” formula. The accuracy of the formula prediction is essential for the successful application of the semi-quantification methodology. In a previous study, the algorithm for formula prediction in the MZmine 2 framework was tested across 48 chemicals with the observation that 79% of compounds were predicted correctly as the highest-ranking candidate, i.e., the lowest difference in ppm.^50^ In this study, 12066 features were identified in total across the wood burning extracts, with the highest ranking candidate accepted as the “best” formula for 97.6% of features. Furthermore, the possibility that a CHO species could be mistakenly predicted as a CHON compound was minimal given the odd mass of odd nitrogen species and the isotope fitting applied in the MZmine workflow. However, in the 443 cases where a CHO species had a CHON compound as the second ranking candidate which occurred exclusively for C_*x*_H_*y*_O_5_ compounds, the second candidate was a C_*x*_H_*y*_N_4_O compound. This is a highly unlikely combination of heteroatoms to be observed in organic aerosol; therefore, formula misidentifications were considered to be minimal in this work. Blank subtraction involved three steps: (1) common species detected in the sample and filter blank or chamber blank were removed if the sample-to-filter-blank signal was <10 or the sample-to-chamber-blank signal ratio was <10 to ensure removal of all false positive peaks; (2) the 20 most abundant surfactants and chlorinated organonitrate compounds in the chamber background, not removed in the first step due to large signals, were also removed from the sample owing to poor chromatography; and (3) only species with a signal-to-noise ratio >3 were accepted. In the final step (iii), species which also ionized in positive mode were retained only in negative mode analysis if better ionization, i.e., a larger peak area, was achieved. This step, although not crucial for this work as the semi-quantification method was developed in negative mode, enables positive and negative mode to be merged in a future analysis where ideally NTA covers both compositional spaces. This workflow was applied to the wood burning aerosol extracts in order to evaluate its performance compared to traditional peak area methods frequently used in organic aerosol analysis. A total of 389–2357 features were detected by the NTA across the different samples, where variation in the feature detection is largely due to variability in filter mass concentration.

### Evaluation of Matrix Effects and Recovery

While the standard solutions used to construct the methodology were analyzed in pure solvent, matrix effects can arise when in the wood burning sample matrix^51,52^ leading to the enhancement or suppression of the peak signal. This can result in over- or under-estimations of species concentration. Twenty-seven species were structurally identified to Schymanski Level 1^53^ in the wood burning samples; therefore, the matrix effect was evaluated for these compounds with a linear standard addition calibration curve (R^2^ ≥ 0.8) (Table S4). The experimental procedure for the determination of matrix effects is explained in detail in the Supporting Information. The matrix effect is expressed as the ratio of the internal standard to the external standard calibration gradient for each compound. On average, we report a relatively low gradient matrix effect of 0.864 ± 0.442 which is likely accounted for within the uncertainty of the semi-quantification method. The ratios of the externally calibrated concentrations to the standard addition calibration were also calculated with a mean average of 0.925 ± 0.475. Using the classification adopted for quality control and method validation for pesticide analysis as there is yet a universal quality assurance and control framework to exist for organic aerosol,^54^ the calculated matrix effects are within the accepted range of ±20% suppression or enhancement^55−58^ and comparable to a previous organic aerosol study.^59^ Nonetheless, in a nontarget analysis where the majority of compounds are unknown it is impossible to quantify an exact matrix effect for each compound, and using a surrogate internal standard cannot fully compensate for the analytical variation.^60^ Furthermore, the extraction recovery of an analyte from organic aerosol collected on a filter can be challenging to exactly replicate in a laboratory, as this involves the recovery from a matrix adsorbed onto a second matrix. Instead, the recovery of the 27 identified compounds was approximated from spiking standards at known concentration onto a blank filter resulting in an average recovery of 88.5 ± 3.9% (Table S4). The relative standard deviation of the individual recoveries in Table S4 were less than 20% and therefore are considered satisfactory.^61^

## Results and Discussion

### Development of the Semi-Quantitative Approach

To overcome differences in ionization efficiencies, calibration using authentic standards is required. However, in a complex sample containing thousands of unknown species the lack of commercially available authentic standards means that accounting for ionization efficiency is practically impossible. Instead, a semi-quantification approach can be used wherein calibration gradients from proxy standards are applied to unknown species. Calibration gradients for oxygenated (CHO), organonitrate (CHON), and organosulfate (CHOS) species were obtained across a 7 point calibration curve for each analytical standard as described in Instrument and Data Analysis. Concentrations were analyzed in triplicate, and the linear fit was not forced through 0. A total of 110 standards were used in total: 90 predominantly organoacids and alcohols for the CHO class, 19 nitroaromatic standards for the CHON compounds, and the use of camphorsulfonic acid for CHOS species. Due to the operation of the ESI source in negative mode, the chosen standards were expected to ionize favorably under these conditions. The standards and their corresponding gradients are presented in Table S1; however, for the purpose of this study, *y*-axis intercepts were ignored.

The acquired chromatogram from the UHPLC-HRMS method was split into retention time windows, assigning each authentic standard to a retention time window, as shown in Table S1. For CHO, the number of standards allowed retention time windows of 1 min from 0–14 min and windows of 2 min from 16–20 min, resulting in 17 retention time windows. For CHON, retention time windows range between 2 and 3 min due to the lower number of available standards, resulting in 8 retention time windows (Table S1). A scaling factor was obtained for each retention time window by calculating the median calibration gradient across the authentic standards within each retention time window. To allow for estimates of uncertainty, the lower quartile, upper quartile, and minimum and maximum calibration gradients were also computed per retention time window. For those compounds identified by the spectral library to Schymanski Level 1,^53^ scaling is achieved using the authentic standard calibration gradient. For unidentified compounds, the chromatogram is split into the retention time windows described above and scaled with the corresponding averaged retention time window calibration gradient to enable the semi-quantification of all CHO and CHON species (see Table S5) detected by the NTA. The overview of this strategy is presented in Figure S1.

### Validation of the Semi-Quantification Method

**Figure 1:**
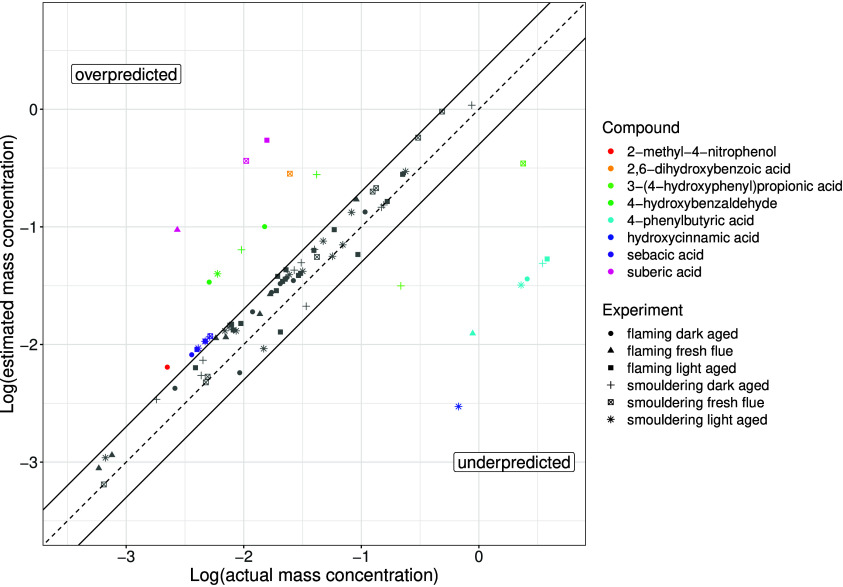
Comparison of the semi-quantification method (*y* axis) with authentic standard quantification (*x* axis) for the estimated concentration (μg m^–3^) of identified compounds present within the different wood burning aerosol samples (markers). The 1:1 line is presented as a dashed line, and the 1:2 and 2:1 lines are indicated by the solid lines. Compounds within this prediction range (factor of 2) are shown as gray markers with the outlying compounds presented in colors. Different wood burning samples are indicated by the marker symbol.

The semi-quantification method was applied to a series of different biomass burning aerosol extracts. In order to determine the performance of the semi-quantification method, the semi-quantified concentrations of structurally identified compounds were compared to quantification using authentic standards. Quantification errors were determined from averaging (here we use the median), the ratio of a species concentration estimated via the semi-quantification method to the concentration determined with an authentic standard (eq 1) and therefore are represented as an error of n times compared to the concentration determined using an authentic standard. The concentrations used in eq 1 were not subject to logarithmic transformations, and ratios greater than and less than 1 were included in this calculation. Of the 27 structurally identified species detected in the wood burning samples, 70% of the concentrations determined by the semi-quantification method were within a factor of 2 of the authentic standard derived concentration, and these compounds are shown in gray in Figure 1, suggesting that the majority of compound concentrations are accurately estimated by the semi-quantification method. The compounds outside of this error range are shown in color in Figure 1. It is important to note that due to the use of the median gradient for each retention time window some of the identified compounds may be scaled with their own authentic standard gradient, more likely for the CHON species due to the smaller number of surrogate standards, and therefore sit on the 1:1 line in Figure 1.
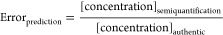
1

McCord et al.^27^ similarly demonstrated a low prediction bias (<48%) for the quantification of emerging perfluoroethercarboxylic acids (PFECAs) in drinking water using 4 different surrogate PFECAs standards which eluted within a 4 min retention time window of the unknown PFECAs. However, the method validation for the semi-quantification was applied only to a single known compound; therefore, McCord et al.^27^ estimated prediction errors of up to 10-fold for the unknown emerging PFECAs. Prediction errors of 1.74 times and 3.20 times compared to quantification by authentic standards were observed by Pieke et al.^42^ and Kruve et al.,^38^ respectively, for the quantification of unknown compounds in food and groundwater analysis using closely eluting markers, typically ±2 min. Comparatively, the semi-quantification method developed in this work had a median prediction error of 1.52 and a mean prediction error of 3.14 times across 27 structurally identified compounds showing improved prediction accuracy when using more than a single proxy standard for quantification. The prediction error is lower for CHON species (1.32) than for CHO compounds (1.52). This is in contrast to the quantification error observed in the predictive ionization efficiency model developed by Sepman et al.^22^ with larger prediction errors in CHON quantification ranging up to a factor of 10. However, the model was developed for positive mode ESI; therefore, the analyzed compounds likely possess different functionality from the CHON species presented in this work.

For the species predicted outside of the factor of 2 error range, the uncertainty in concentration, calculated using the interquartile range of calibration gradients in each retention time window was compared to quantification by an authentic standard (Figure S2). This showed improvements in 3 outlying species: sebacic acid, 4-phenylbutyric acid, and 3-(4-hydroxyphenyl)propionic acid. The remaining outliers typically have gradients at the extremities of their corresponding retention time window. The outliers were not correlated with retention time and therefore were assumed to be little affected by the increasing organic modifier content of the mobile phase. Furthermore, outliers were present at multiple retention times throughout the chromatography runtime, indicating that species polarity is also not a determining factor. Instead, they could result from other structural properties affecting ionization efficiency, including the p*K*_a_ and molecular weight of a species.^11,13^ This influences the interactions of the analytes with the solvent droplet and the ease of deprotonation in negative mode ESI. In Figure 1, overprediction resulted from scaling with a lower gradient compared to the authentic slope, whereas underprediction occurred from scaling using larger gradients than the authentic slope. p*K*_a_, which governs the ability to deprotonate, may affect the overprediction of 2,6-dihydroxybenzoic acid concentrations. For instance, 2,6-dihydroxybenzoic acid possessed the highest ionization efficiency of the compounds within its retention time window while simultaneously having the lowest p*K*_a_, predicted by ChemDraw 21.0.0 software, which suggests a greater deprotonation ability compared to other species within the same window. On the other hand, multiple ionization sites as in suberic acid, a dicarboxylic acid, could increase the ionization efficiency compared to the monocarboxylic acids within the same retention time window. In addition to p*K*_a_ and the number of deprotonation sites, stabilization of the deprotonated ion further affects the ionization efficiency. For instance, despite ionization at a higher p*K*_a_ alcohol group, the deprotonated 4-hydroxybenzaldehyde ion could exhibit charge stabilization effects, thereby increasing its ionization efficiency compared to the other compounds within the same window. However, further work is needed to investigate these effects and if they can be accounted for.

Due to the nature of NTA, the chemical functionality present within a sample can be difficult to predict; therefore, a wide range of standards of different chain length, aromaticity, and functionality were used in this study (Table S1). As such, retention time windows can have large variations in calibration gradients between the different surrogate standards owing to the molecular properties previously discussed. For instance, the retention time window which overpredicted 2,6-dihydroxybenzoic acid concentrations had the maximum observed difference of 4 orders of magnitude between the maximum (2,6-dihydroxybenzoic acid) and minimum (butyric acid) gradient. Removing the butyric acid gradient from this window decreased the difference in gradients to 3 orders of magnitude, and the overprediction of 2,6-dihydroxybenzoic acid was reduced from 11 times to 6 times compared to quantification by authentic standard. Therefore, prior chemical knowledge of the sample could improve quantification through the selection of targeted standards which better reflect the sample composition. However, due to the lack of commercially available authentic standards, this approach is not always possible.

### Comparison to an Existing Machine Learning Predictive Model

In a number of previous studies, ionization efficiency has been predicted from machine learning models, based on either physicochemical properties, structural descriptors, or chemical fingerprints.^17−22,62^ Bryant et al.^18^ built a model based on chemical structural fingerprints obtained from the ChemDes platform^63^ to predict RIEs of 89 CHO and CHON compounds using *cis*-pinonic acid as the reference compound for the quantification of biogenic SOA markers. It is worth noting that this method required prior knowledge of the structure in order to predict the RIE and therefore is not applicable to unknown compounds. More recently, molecular descriptors from MS^2^ have been used to predict the ionization efficiency, which could yield further improvements in quantification for structurally unidentified compounds.^22^ However, for species without MS^2^, quantification remains a challenge. RIE predictions were taken from Bryant et al.^18^ and applied to the wood burning samples to determine the concentration of 18 structurally identified compounds that were quantifiable by both methods. Figure S3 shows the comparison of the concentration predicted by the semi-quantification approach developed here with the RIE predictions from Bryant et al.^18^ for estimating the concentrations of structurally identified compounds within the wood burning extracts. Comparisons to other existing predictive models are difficult due to the use of different LC methodologies which could induce additional ionization effects from the solvent system as well as the use of different reference compounds for calculating RIE.^18−20^ However, future work should aim to include interlaboratory comparisons when applying the same methodology to ensure that the performance is consistent as recently demonstrated by Malm et al.^64^ across 37 laboratories. Of the 18 common compounds quantified by both methods, a third were semi-quantified to within a factor of 2 compared to quantification with Bryant et al.^18^ RIE predictions including sebacic acid, azelaic acid, 3-methyl adipic acid, adipic acid, glutaric acid, and succinic acid. A further 6 organoacid species could be semi-quantified to within a factor of 2 of the RIE method by applying the semi-quantification method’s uncertainty range, calculated from the interquartile range of concentration. Overall, this indicated good agreement between the methods for the quantification of CHO compounds but greater discrepancy for the quantification of CHON which was further away from the factor of 2 prediction errors lines in Figure S3.

**Figure 2:**
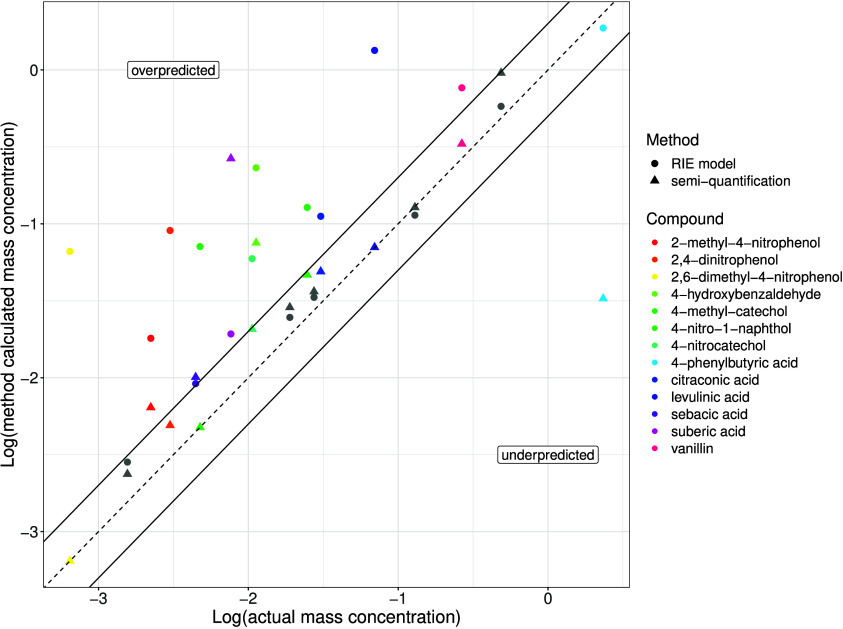
Comparison of semi-quantification (triangle markers) and RIE predictive model (circle markers) methodologies (*y* axis) with authentic standards (*x* axis) for the quantification of identified compounds, shown as average concentrations (μg m^–3^), within the wood burning aerosol samples. The 1:1 line is presented as a dashed line, and the 1:2 and 2:1 lines are indicated by the solid lines. Compounds within a factor of 2 from the authentic standard concentration in both methods are shown as gray markers. Compounds which do not meet this condition are presented in color.

Compared to quantification by authentic standards, the predicted RIEs from Bryant et al.^18^ tended to overpredict the species concentration compared to using the semi-quantification methodology (Figure 2) as a result of underpredicting the RIE. The compounds in gray in Figure 2 represent a low prediction error, i.e., less than a factor of 2, in both methods compared to quantification with authentic standards and were mostly compounds with good agreement between the methods (Figure S3). The majority of the compound concentrations estimated by the semi-quantification method in Figure 2 were more closely situated to within a factor of 2 of the concentration determined using authentic standards, which demonstrated improved prediction errors compared to the RIE methodology. However, significant exceptions exist for suberic acid and 4-phenylbutyric acid with prediction errors compared to quantification by an authentic standard of 34.72 and 0.01 (or 100 times lower), respectively, using semi-quantification compared to 2.52 and 0.80 (or 1.25 times lower), respectively, using RIE predictions. Overall, the CHO compounds had a median prediction error of 1.52 and 2.05 for the semi-quantification and RIE predictive model approaches, respectively, showing similar performance between the methods for estimating concentration compared to using authentic standards. The estimation of nitroaromatic compound concentrations was less certain using the RIE approach with median prediction errors of 14.94 times compared to quantification by authentic standard; however, the RIE model developed by Bryant et al.^18^ underrepresents nitroaromatic compounds in the training data, leading to an underprediction of their RIE. The semi-quantification method used a similar number of nitroaromatic compounds to create the retention time windows and had a lower prediction error of 1.63 times for the same compounds compared to quantification by authentic standard. Furthermore, in the interlaboratory study by Malm et al.^64^ they observed that semi-quantification using singular close eluting standards performed worse compared to RIE model approaches. Therefore, the semi-quantification approach developed here using multiple close eluting standards shows that choosing suitable retention time windows even with a relatively small number of standards can be a more effective method to improve quantification, yielding similar or more accurate concentrations than RIE predictive model approaches.

### Application of Nontarget Analysis to Biomass Burning Aerosol Samples

**Figure 3:**
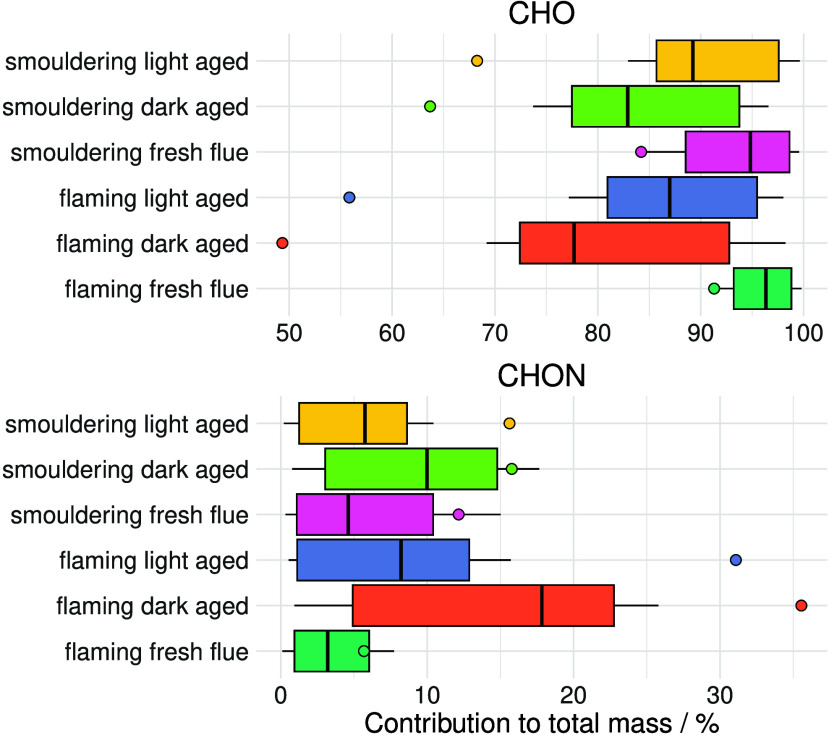
Percentage contribution of CHO and CHON compounds to the total mass concentration within laboratory-generated wood burning aerosol, colored by different conditions. The box plot represents the relative abundance determined from quantification using the median, lower quartile, upper quartile, minimum, and maximum calibration gradients for each compound in each retention time window. The points represent the relative abundance derived using peak area.

This semi-quantitative nontarget methodology was designed for use in highly chemically complex samples such as that found in an atmospheric organic aerosol derived from biomass burning, owing to the sheer number and functionality of compounds present, meaning that quantification is challenging. However, the general methodology framework of using multiple retention time windows with numerous chemically relevant standards can be applied to other chemically complex environmental and biological matrices. The chemical composition of BBOA is highly dependent on fuel type, burning conditions, and atmospheric aging, resulting in a large variation and degree of complexity.^65−67^ Application of the semi-quantification method enabled distinct differences in the bulk composition, through the relative ratio of CHO:CHON contributions, to be observed between different burn phases and aging processes (Figure 3). In Figure 3, the relative abundance was derived from the quantification of each compound using the median, lower quartile, upper quartile, maximum, and minimum calibration gradient for their corresponding retention time window. The uncertainty of the method for estimating the relative abundance was then derived from the interquartile range of abundance shown in Figure 3. Across the BBOA samples, the average uncertainties in relative abundance, determined from the interquartile range in Figure 3, were 12.8% and 10.2% for CHO and CHON species, respectively. Depending on the metric used to estimate abundance in NTA, the overall compositional contributions can vary, leading to differences in source apportionment. For instance, as shown in Figure 3, on average CHO compounds contribute 88.1 (± 7.1)% to the total mass using the semi-quantification method or 68.8 (± 16.2)% of the total peak area. Therefore, using peak area to determine abundance underestimated the contribution of CHO species to the total BBOA mass by 19 (± 10)%. The compounds in the CHON group contribute 8.2 (± 5.2)% or 19.3 (± 11.5)% using semi-quantification and peak area, respectively, resulting in an overprediction of 11 (± 8)% on average. Furthermore, the difference between the methods for estimating abundance can reach 31% depending on the sample (see Table S6).

CHON species from biomass burning have largely been assigned as nitroaromatic compounds and are widely used as tracers for biomass burning in ambient aerosol due to their conceived high abundance^6,9^ and important implications for atmospheric brown carbon (BrC).^6,68,69^ However, this study determined the average relative abundance of CHON to be 8.2%, which is lower than that estimated if using peak area, suggesting that peak area can significantly overestimate the contribution of CHON to BBOA. Instead, the semi-quantification method found a significant contribution of CHO (>85%) to BBOA, indicating that CHO species could be important tracers of biomass burning. Furthermore, these differences in the estimation of the relative abundance of each compound when using semi-quantification or peak area can be propagated into metrics commonly used to characterize organic aerosol composition and atmospheric oxidation such as the average molecular formula and oxygen:carbon ratios (Table S7).

## Conclusions

A semi-quantitative approach to estimate concentrations of unidentified compounds was developed for use within NTA workflows of complex samples, such as organic aerosol, analyzed by UHPLC-HRMS. The method used retention time windows to derive unique scaling factors from multiple authentic standards for each defined window. The total quantification of chemical space is improved compared to existing predictive ionization efficiency models due to the lack of a requirement to know the structure or have access to fragmentation mass spectra. The method was validated against 27 structurally identified species, quantified using authentic standards, in a range of BBOA extracts from wood burning with an overall average prediction error, defined as the ratio of concentrations determined with the semi-quantification method to that using an authentic standard, of 1.52. This improved upon previous semi-quantification methods using closely eluting quantification markers which yield errors of up to one order of magnitude. Compared to a predictive ionization efficiency model, the semi-quantification method demonstrated improved performance for the quantification of nitroaromatic species despite using a similar number of authentic standards. Comparison of the semi-quantification method to widely used peak area approaches in NTA highlighted the inadequacy of using peak area to calculate relative abundance in complex sample analysis, with differences in abundance reaching 31% among the different methods. This represents a significant potential to misinterpret source apportionment contributions. Future work is needed to fully comprehend matrix effects in highly complex samples and apply the method to positive mode ionization for complete quantification. Overall, we highlight the need to standardize nontarget quantification metrics and suggest utilizing the semi-quantification method independently or in combination with existing predictive ionization efficiency models to create a robust NTA workflow of all (MS^1^ and MS^2^) detected features for application in complex sample analysis.

## Supporting Information Available

Standards and retention time windows used and all detected features for quantification (XLSX)
Description of filter extraction method; UHPLC-HRMS parameters; matrix effects and extraction recovery results; semi-quantification workflow; MZmine parameters; uncertainty of semi-quantified concentrations with authentic standards; method comparison with a predictive ionization efficiency model; method comparison with peak area; comparison of average aerosol NTA metrics (PDF)
