# Supplementary material for: A Semi-Quantitative Approach to Nontarget Compositional Analysis of Complex Samples
Source: Anal Chem. 2024 Nov 7;96(46):18349–58. doi: 10.1021/acs.analchem.4c00819 (PMC11579983; doi:10.1021/acs.analchem.4c00819)
Supplement: Supplementary file 2 — ac4c00819_si_002.pdf [file ac4c00819_si_002.pdf]

Supporting Information
A semi-quantitative approach for non-target compositional analysis of complex samples

Rhianna L. Evans,[†] Daniel J. Bryant,[†] Aristeidis Voliotis,[‡,¶] Dawei Hu,[‡] HuiHui Wu,[‡] Sara Aisyah Syafira,[‡] Osayomwanbor E. Oghama,[‡] Gordon McFiggans,[‡] Jacqueline F. Hamilton,[*,†,§] and Andrew R. Rickard[*,†,§]

†*Wolfson Atmospheric Chemistry Laboratories, Department of Chemistry, University of York, York, YO10 5DD, United Kingdom*

‡*Centre for Atmospheric Science, Department of Earth and Environmental Sciences, School of Natural Sciences, University of Manchester, Manchester, M13 9PL, United Kingdom*

¶*National Centre for Atmospheric Science, University of Manchester, Manchester, M13 9PL, United Kingdom*

§*National Centre for Atmospheric Science, University of York, York, YO10 5DD, United Kingdom*

E-mail: jacqui.hamilton@york.ac.uk; andrew.rickard@york.ac.uk

Contents

1 Filter Extraction **S3**

2 UHPLC-HRMS methodology **S4**

3 Determination of matrix effects and recovery **S5**

List of Figures

S1 Semi-quantification workflow S7

S2 Uncertainty of the semi-quantification methodology for quantification S8

S3 Comparison to an existing predictive RF model S9

List of Tables

Tables provided as .xlsx

S1 Authentic standards and retention time windows used in the semi-quantification methodology S2

S5 Semi-quantified CHO and CHON features S2

Tables in this document

S2 MZmine 2.53 workflow S10

S3 MZmine 3.9.0 workflow S11

S4 QA/QC of the semi-quantification method S12

S6 Differences in relative abundance between peak area and semi-quantification S13

S7 Average aerosol metrics determined by semi-quantification and peak area . . S14

1 Filter Extraction

The 47 mm quartz filters from the chamber experiments were cut into 1 cm^2 pieces, placed in a 20 mL glass vial and 10 mL of methanol (LC-MS Optima Grade) was added. However for fresh samples, half a 47 mm filter was used due to their higher mass concentration. The resulting solution was sonicated for 45 minutes, using ice packs to lower the temperature of the water bath. The methanol extract was transferred to a second 20 mL glass vial using a 0.22 μm syringe filter (Milipore) then dried using a Genevac vacuum solvent evaporator. The sample was reconstituted in 200 μL 90:10 H_2O (LC-MS Optima Grade): MeOH (LC-MS Optima Grade) for UHPLC-HRMS analysis.

2 UHPLC-HRMS methodology

Compound separation was achieved using a reversed phase C_{18} 2.6 μm x 2.1 mm x 10 mm Accucore column held at 40 °C. The mobile phase consisted of 0.1 % (v/v %) formic acid (Acros Organics) in water (A, LC-MS Optima Grade) and methanol (B, LC-MS Optima Grade). A gradient elution was used, starting at 90 % (A) with a 1 minute post injection hold, decreasing to 10 % (A) at 26 minutes before returning to the starting conditions at 28 minutes and a further 2 minute hold to allow for the re-equilibriation of the column. The flow rate was set to 0.3 mL min^{-1}. Prior to analysis samples were stored in an autosampler tray at 4 °C. The injection volume was set to 4 μL, however injection volumes up to 10 μL were used for lower concentration samples. The HESI was operated under the following conditions: a spray voltage of 4 kV, a capillary and auxiliary gas temperature of 320 °C, a sheath gas flow rate of 45 (arb.) and an auxiliary gas flow rate of 10 (arb.) Spectra were acquired in negative and positive mode using data dependent tandem mass spectrometry (ddMS^2). The scan range was set to a mass-to-charge ratio (m/z) of 85 to 750, with a mass resolution of 140,000. Tandem mass spectrometry was performed using a higher collision dissociation with a stepped normalised collision energy of 10, 20 and 45. In each scan the 10 most abundant species were selected for MS^2 fragmentation.

3 Determination of matrix effects and recovery

Using quality control recommendations by Schulze et al.[1] a pooled sample was taken of the wood burning samples to represent an "average" sample matrix effect. Of each sample, depending on availability a 25-75 μL aliquot was taken and combined into a pooled sample (450 μL in total). Due to the low sample availability the matrix effect was determined from a 4 point internal standard calibration 10 μL aliquots of 2.5, 2.0, 1.5 and 1.0 ppm standard solutions of the 27 analytical standards in 90:10 H_2O:MeOH were spiked into four 90 μL aliquots of the pooled sample, giving a 1 in 10 dilution to produce the final calibration concentrations of 250, 200, 150 and 100 parts per billion (ppb). The same volume of stock solution was then spiked into 90μL of 90:10 H_2O:MeOH to produce pure standard solutions at the same concentrations for comparison. Calibration curves of the pure standard solutions and the internal standard solutions were constructed. The peak area of the internal standards was determined from subtracting the pooled sample peak area from the spiked pool sample peak area. The gradient matrix effect was then determined as in Eq. S1 as the ratio of the internal standard to external standard calibration gradient for each compound (Table S4). To further understand the impact on species concentration standard addition calibration curves were constructed from the spiked pooled samples without removing the pooled sample peak area and then extrapolating the line to y = 0 to determine the pooled sample concentration. This concentration was the compared to that determined from solving the external calibration regression line at the pooled sample peak area. The concentration matrix effect reported in Table S4 is expressed as the ratio of the externally calibrated concentration to the standard addition determined concentration (Eq. S2). Average matrix effects were determined from 17 internal calibrations. Only compounds with a $R^2 \geq 0.8$ in the standard addition curve were included to reduce the impact of anomalous compounds with poor linearity. These calibrations more highly perturbed by the matrix and were predominantly compounds which eluted early (< 2 minutes) as seen previously[2] or had low ionisation efficiencies ($\times 10^6$-10^7).

In the external calibrations the R^2 was greater than 0.98 for 26 of the 27 identified compounds therefore the deterioration in linearity can be attributed to the matrix.

$$ME_{\text{gradient}} = \frac{Gradient_{\text{internal}}}{Gradient_{\text{external}}} \tag{S1}$$

$$ME_{\text{concentration}} = \frac{Concentration_{\text{external}}}{Concentration_{\text{internal}}} \tag{S2}$$

Recovery of 27 analytical standards which were identified in the wood burning samples were determined from spiking 50 μL of a 100ppm standard solution for each standard onto a blank filter to achieve an on column concentration of 5ppm or 5 μg mL^{-1}. The spiking procedure was repeated 3 times on 3 separate filters. Each spiked filter and a blank non-spiked filter were extracted according to the methodology described in Section "Filter Extraction". A pure 1mL stock solution of 5ppm of each analyte in 90:10 H_2O:MeOH was made up and analysed by UHPLC-HRMS at the same time as the spiked filters to determine the analyte recovery shown in Table S4, calculated as the percentage of the filter peak area at 5ppm compared to the 5ppm pure standard solution.

Peak detection for the matrix effect and recovery compounds was computed using an accurate mass and retention time library in TraceFinder 4.1 with a retention time window of 30 seconds and a minimum signal-to-noise ratio of 3. For species not detected by the TraceFinder library, the peak area was manually integrated in the acquisition software XCalibur 4.3. For levulinic acid the repeatability of the recovery samples was poor therefore a single sample was taken resulting in the lack of a standard deviation value in Table S4.

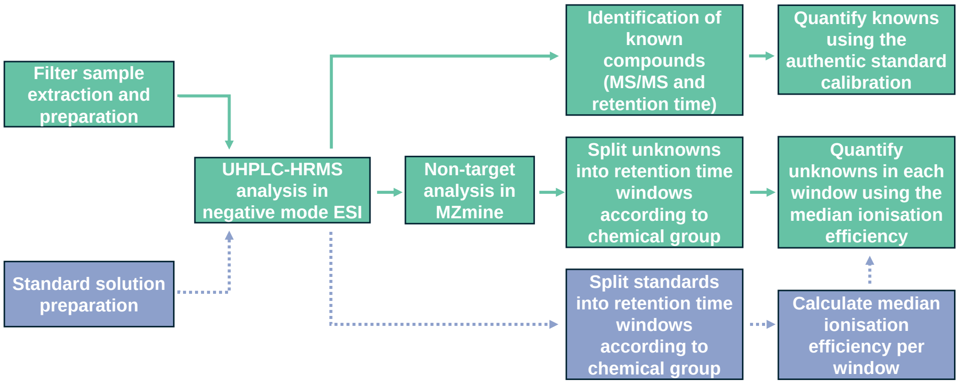

Figure S1: The semi-quantification workflow depicting the scaling of known compounds with authentic standards and unknown species via the retention time window approach.

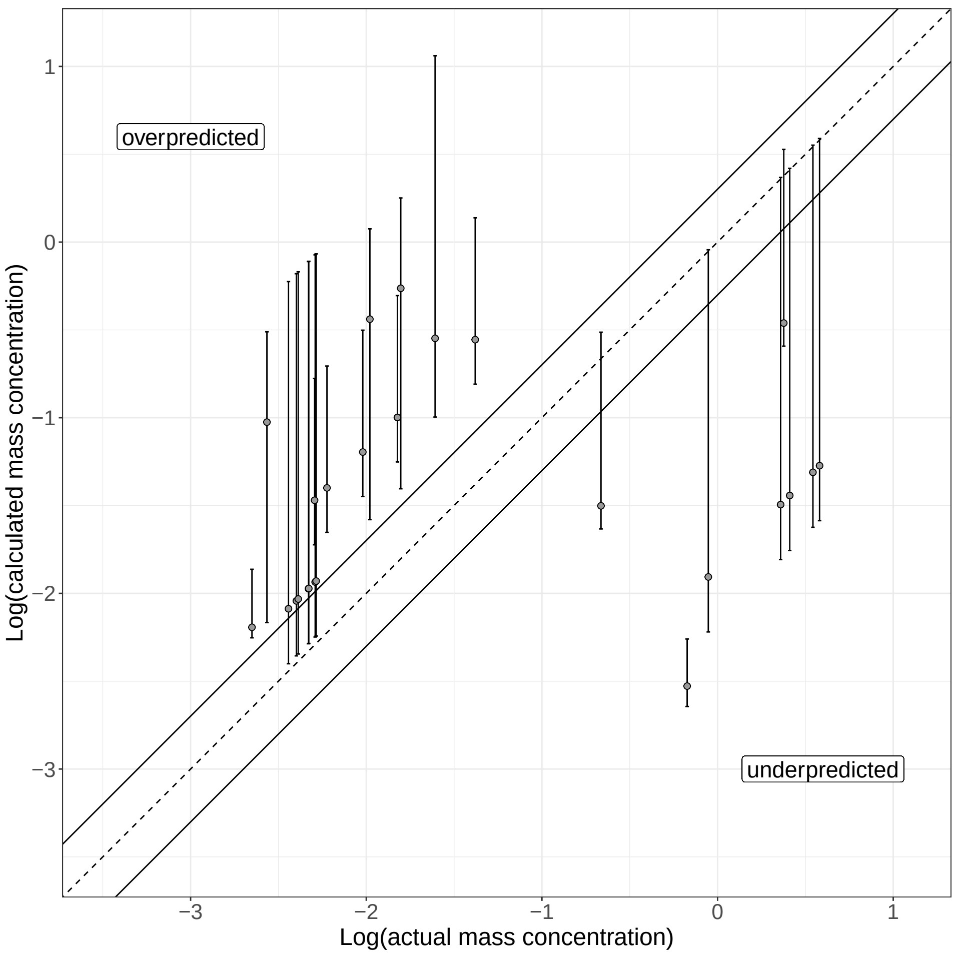

Figure S2: Comparison of the outlying compounds quantified by the semi-quantification method (y-axis) and authentic standard (x-axis) in the wood burning samples. 1:1 line is presented as a dashed line and 1:2 and 2:1 lines are indicated by the solid lines. Points represent median values and error bars depict the interquartile range calculated using the interquartile range of gradients in each retention time window.

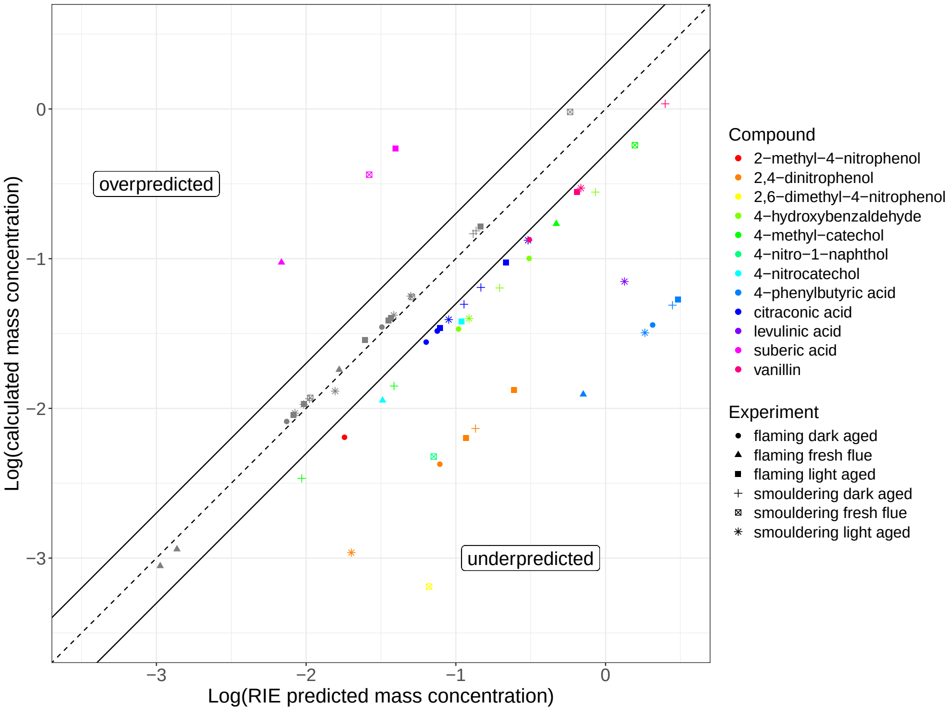

Figure S3: Comparison of the semi-quantification method (y-axis) with the RIE prediction (x-axis) for quantification of identified compounds within the wood burning aerosol samples. 1:1 line is presented as a dashed line and 1:2 and 2:1 lines are indicated by the solid lines. Compounds within this prediction range (factor of 2) are shown as grey markers with the outlying compounds presented in colour. Different wood burning samples are indicated by the marker symbol.

Table S2: Workflow used for the non-target feature detection in MZmine 2.53 and the parameters set for each module

Mass Detection	Retention time = 0 - 20 min<br>MS Level = 1<br>Polarity = -<br>Spectrum type = profile<br>Mass detector = Exact Mass<br>Noise level = 50000
FTMS Shoulder Peaks Filter	Mass resolution = 140,000<br>Peak model function = Lorentzian
Mass Detection	Retention time = 0 - 20 min<br>MS Level = 2<br>Polarity = -<br>Spectrum type = profile<br>Mass detector = Exact Mass<br>Noise level = 0
ADAP Chromatogram Builder	Min group size in # of scans = 5<br>Group intensity threshold = 50000<br>Min highest intensity = 60000<br>m/z tolerance = 0.001 m/z or 3 ppm
Smoothing	Filter width = 7
Chromatogram Deconvolution	Algorithm = local minimum search<br>Chromatographic threshold = 80 %<br>Search minimum in RT = 0.3 min<br>Minimum relative height = 30 %<br>Minimum absolute height = 50000<br>Min ratio of peak top/edge = 1.2<br>Peak duration = 0 - 2 min<br>m/z centre calculation = median<br>m/z range for MS2 scan pairing = 0 Da<br>RT range for MS2 scan pairing = 0.05 min
Join Aligner	m/z tolerance = 0.001 m/z or 3 ppm<br>Weight for m/z = 3<br>RT tolerance = 0.1 min<br>Weight for RT = 1
Isotopic Peaks Grouper	m/z tolerance = 0.001 m/z or 3 ppm<br>RT tolerance = 0.01 min<br>Monotonic shape<br>Maximum charge = 1<br>Representative isotope = most intense
Duplicate Peak Filter	Filter mode = new average<br>m/z tolerance = 0.0008 m/z or 1.5 ppm<br>RT tolerance = 0.01 min
Formula Prediction	Charge = 1<br>Ionisation type = [M-H]-<br>m/z tolerance = 0.001 m/z or 3 ppm<br>Max best formulas per peak = 5<br>Elements = $C_{1\text{-}40}H_{0\text{-}100}O_{1\text{-}10}N_{0\text{-}4}S_{0\text{-}2}Cl_{0\text{-}2}$<br>Element count heuristics Y<br>RDBE restrictions Y

Table S3: Workflow used for the spectral library search in MZmine 3.9.0 and the parameters set for each module

Mass Detection	Retention time = 0 - 26 min<br>MS Level = 1<br>Polarity = -<br>Spectrum type = profile<br>Mass detector = Exact Mass<br>Noise level = 50000
Mass Detection	Retention time = 0 - 26 min<br>MS Level = 2<br>Polarity = -<br>Spectrum type = profile<br>Mass detector = Exact Mass<br>Noise level = 0
ADAP Chromatogram Builder	RT = 0 - 26min<br>MS level = 1<br>Polarity = -<br>Spectrum type = profile<br>Min group size in # of scans = 5<br>Min intensity for consecutive scans = 10000<br>Min highest intensity = 50000<br>m/z tolerance = 0.0008 m/z or 2 ppm
Minimum Search Feature Resolver	Algorithm = local minimum search<br>Chromatographic threshold = 90 %<br>Minimum search range in RT = 0.3 min<br>Minimum absolute height = 50000<br>Min ratio of peak top/edge = 1.5<br>Peak duration = 0 - 2 min<br>Minimum scans = 5<br>MS/MS scan pairing = TRUE<br>MS1 to MS2 precursor tolerance = 0.01 m/z or 10ppm
Isotopic Peaks Grouper	Chemical elements = C,H,O,N,S,Cl<br>m/z tolerance = 0.0005 m/z or 10 ppm<br>Maximum charge = 1<br>Search in scans = single most intense
Join Aligner	m/z tolerance = 0.0008 m/z or 2 ppm<br>Weight for m/z = 3<br>RT tolerance = 0.1 min<br>Weight for RT = 1
Spectral Library Search	Scans for matching = MS level $\geq$ 2 (Merged)<br>Precursor m/z tolerance = 0.001 m/z or 5 ppm<br>Spectral m/z tolerance = 0.0015 m/z or 10 ppm<br>Minimum matched signals = 4<br>Similarity = weighted cosine similarity<br>Minimum cos similarity = 0.7<br>Handle unmatched signals = keep all and match to 0<br>RT tolerance = 0.25 min

Table S4: QA/QC of the semi-quantification method showing the percentage analyte recovery from the filter extraction, relative standard deviation (RSD), matrix effects on the calibration slopes, matrix effect on the sample concentration and the retention time variability for the identified species in the wood burning extracts

Compound	Extraction recovery / %	RSD / %	Gradient matrix effect	Concentration matrix effect	RT variability / min
2,6-dihydroxybenzoic acid	104.4 ± 2.9	2.7	-	-	-
2,6-dimethyl-4-nitrophenol	75.8 ± 3.2	4.2	-	-	-
2-4-dinitrophenol	80.7 ± 11.1	13.8	-	-	0.15
2-methyl-4-nitrophenol	92.5 ± 12.3	13.3	0.759	0.557	-
3,4,5-trimethoxybenzoic acid	94.0 ± 4.2	4.4	-	-	0.02
3,4-dihydroxybenzoic acid	91.4 ± 3.1	3.4	0.943	0.927	-
3,5-dimethoxy-4-hydroxybenzoic acid	80.5 ± 2.5	3.0	0.850	0.994	-
3-(4-hydroxyphenyl)propionic acid	85.3 ± 10.2	11.9	-	-	0.12
3-hydroxy-2-napthoic acid	82.5 ± 1.5	1.8	0.762	0.145	-
3-methyladipic acid	76.2 ± 2.3	3.1	0.914	1.658	0.06
4-hydroxybenzaldehyde	80.9 ± 0.5	0.7	0.644	1.254	0.09
4-methyl-catechol	62.5 ± 10.2	16.3	0.708	0.770	0.17
4-nitro-1-napthol	61.6 ± 4.3	7.0	-	-	-
4-nitrocatechol	96.5 ± 8.4	8.7	0.449	0.559	0.05
4-nitroguaiacol	74.6 ± 3.2	4.3	-	-	0.10
4-phenylbutyric acid	82.8 ± 7.1	8.5	-	-	0.33
adipic acid	94.6 ± 1.8	1.9	0.145	0.116	-
azelaic acid	90.9 ± 5.2	5.8	0.754	0.732	0.17
citraconic acid	97.5 ± 11.2	11.5	2.260	1.870	0.28
glutaric acid	110.5 ± 14.6	13.2	1.410	0.960	0.15
hydroxycinnamic acid	86.4 ± 3.9	4.5	0.785	0.837	-
levulinic acid	146.8	-	0.957	1.485	-
methylsuccinic acid	97.7 ± 6.7	6.8	-	-	0.25
sebacic acid	95.2 ± 2.3	2.4	0.905	1.271	0.14
suberic acid	100.8 ± 4.0	4.0	0.660	0.743	0.15
succinic acid	92.5 ± 4.8	5.2	-	-	-
vanillin	54.4 ± 6.7	12.3	0.781	0.861	0.22

Table S6: Difference in the relative abundance (%) calculated using peak area and the semi-quantification method for each biomass burning aerosol sample

Compound type	Sample	Method difference
CHO	Flaming fresh flue	-5.1
CHON	Flaming fresh flue	2.5
CHO	Flaming dark aged	-28.5
CHON	Flaming dark aged	17.9
CHO	Flaming light aged	-31.2
CHON	Flaming light aged	22.9
CHO	Smouldering fresh flue	-10.7
CHON	Smouldering fresh flue	7.6
CHO	Smouldering dark aged	-19.3
CHON	Smouldering dark aged	5.9
CHO	Smouldering light aged	-21.0
CHON	Smouldering light aged	9.9

Table S7: Comparison of the semi-quantification method and peak area to determine average metrics commonly used in the non-target analysis of organic aerosol, including, oxygen:carbon ratio, hydrogen:carbon ratio, molecular formula and relative abundance to total mass.

		**Semi-quantification**				**Peak area**			
Sample type	Category	O:C	H:C	Formula	Relative abundance / %	O:C	H:C	Formula	Relative abundance / %
Flaming fresh flue	CHO	0.28	1.05	$C_{12.8}H_{13.0}O_{3.3}$	96.4	0.33	1.09	$C_{11.4}H_{11.9}O_{3.3}$	91.3
Flaming dark aged	CHO	0.38	1.33	$C_{10.4}H_{13.8}O_{3.5}$	77.8	0.47	1.34	$C_{8.5}H_{11.1}O_{3.5}$	49.3
Flaming light aged	CHO	0.42	1.39	$C_{10.1}H_{14.6}O_{3.8}$	87.0	0.47	1.31	$C_{8.7}H_{11.6}O_{3.9}$	55.9
Smouldering fresh flue	CHO	0.35	1.04	$C_{10.6}H_{10.4}O_{3.3}$	94.9	0.40	1.09	$C_{9.6}H_{10.0}O_{3.5}$	84.2
Smouldering dark aged	CHO	0.45	1.45	$C_{9.4}H_{13.0}O_{3.6}$	83.0	0.50	1.44	$C_{8.4}H_{11.5}O_{3.7}$	63.7
Smouldering light aged	CHO	0.49	1.63	$C_{9.4}H_{15.4}O_{3.9}$	89.3	0.53	1.53	$C_{8.1}H_{12.3}O_{3.8}$	71.9
Flaming fresh flue	CHON	0.54	1.74	$C_{13.5}H_{23.1}O_{5.2}N_{1.6}$	3.2	0.47	1.44	$C_{11.5}H_{17.6}O_{4.6}N_{1.2}$	5.7
Flaming dark aged	CHON	0.32	1.43	$C_{14.6}H_{23.2}O_{4.2}N_{1.4}$	17.7	0.42	1.36	$C_{10.9}H_{16.6}O_{4.0}N_{1.3}$	35.6
Flaming light aged	CHON	0.41	1.25	$C_{11.2}H_{14.5}O_{4.3}N_{1.5}$	8.2	0.47	1.03	$C_{8.7}H_{9.5}O_{3.7}N_{1.3}$	32.1
Smouldering fresh flue	CHON	0.42	1.18	$C_{10.4}H_{12.3}O_{3.7}N_{1.3}$	4.6	0.47	1.05	$C_{8.8}H_{9.4}O_{3.8}N_{1.1}$	12.1
Smouldering dark aged	CHON	0.35	1.35	$C_{13.7}H_{18.6}O_{3.8}N_{2.1}$	9.9	0.39	1.30	$C_{11.3}H_{15.6}O_{3.8}N_{1.5}$	15.8
Smouldering light aged	CHON	0.46	1.39	$C_{11.1}H_{15.9}O_{4.7}N_{1.7}$	5.7	0.51	1.24	$C_{9.1}H_{12.0}O_{4.1}N_{1.4}$	15.6

References

(1) Schulze, B.; Jeon, Y.; Kaserzon, S.; Heffernan, A. L.; Dewapriya, P.; O'Brien, J.; Ramos, M. J. G.; Gorji, S. G.; Mueller, J. F.; Thomas, K. V.; Samanipour, S. An assessment of quality assurance/quality control efforts in high resolution mass spectrometry non-target workflows for analysis of environmental samples. *TrAC, Trends Anal. Chem.* **2020**, *133*, 116063.

(2) Williams, M. L.; Olomukoro, A. A.; Emmons, R. V.; Godage, N. H.; Gionfriddo, E. Matrix effects demystified: Strategies for resolving challenges in analytical separations of complex samples. *J. Sep. Sci.* **2023**, *46*, 2300571.
